# Electron Diffraction Using Transmission Electron Microscopy

**DOI:** 10.6028/jres.106.051

**Published:** 2001-12-01

**Authors:** Leonid A. Bendersky, Frank W. Gayle

**Affiliations:** National Institute of Standards and Technology, Gaithersburg, MD 20899-8554

**Keywords:** crystal structure, crystallography, defects, electron diffraction, phase transitions, quasicrystals, transmission electron microscopy

## Abstract

Electron diffraction via the transmission electron microscope is a powerful method for characterizing the structure of materials, including perfect crystals and defect structures. The advantages of electron diffraction over other methods, e.g., x-ray or neutron, arise from the extremely short wavelength (≈2 pm), the strong atomic scattering, and the ability to examine tiny volumes of matter (≈10 nm^3^). The NIST Materials Science and Engineering Laboratory has a history of discovery and characterization of new structures through electron diffraction, alone or in combination with other diffraction methods. This paper provides a survey of some of this work enabled through electron microscopy.

## 1. Introduction

The use of electron diffraction to solve crystallographic problems was pioneered in the Soviet Union by B. K. Vainshtein and his colleagues as early as the 1940s [1]. In the elektronograf, magnetic lenses were used to focus 50 keV to 100 keV electrons to obtain diffraction with scattering angles up to 3° to 5° and numerous structures of organic and inorganic substances were solved. The elektronograf is very similar to a modern transmission electron microscope (TEM), in which the scattered transmitted beams can be also recombined to form an image. As the result of numerous advances in optics and microscope design, modern TEMs are capable of a resolution of 1.65 Å for 300 kV (and below 1 Å for 1000 kV) electron energy-loss combined with chemical analysis (through x-ray energy and electron-loss energy spectroscopy) and a bright coherent field emission source of electrons.

**Fig. 1:**
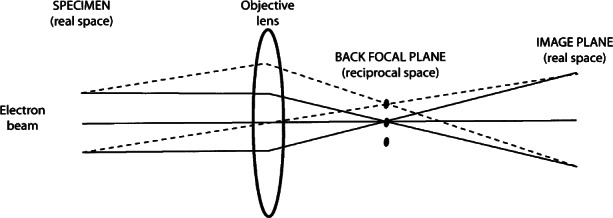
Optical ray diagram with an optical objective lens showing the principle of the imaging process in a transmission electron microscope.

The main principles of electron microscopy can be understood by use of optical ray diagrams [2,3], as shown in Fig. 1. Diffracted waves scattered by the atomic potential form diffraction spots on the back focal plane after being focused with the objective lens. The diffracted waves are recombined to form an image on the image plane. The use of electromagnetic lenses allows diffracted electrons to be focused into a regular arrangement of diffraction spots that are projected and recorded as the electron diffraction pattern. If the transmitted and the diffracted beams interfere on the image plane, a magnified image of the sample can be observed. The space where the diffraction pattern forms is called reciprocal space, while the space at the image plane or at a specimen is called real space. The transformation from the real space to the reciprocal space is mathematically given by the Fourier transform.

A great advantage of the transmission electron microscope is in the capability to observe, by adjusting the electron lenses, both electron microscope images (information in real space) and diffraction patterns (information in reciprocal space) for the same region. By inserting a selected area aperture and using the parallel incident beam illumination, we get a diffraction pattern from a specific area as small as 100 nm in diameter. The recently developed microdiffraction methods, where incident electrons are converged on a specimen, can now be used to get a diffraction pattern from an area only a few nm in diameter. Convergent beam electron diffraction (CBED) uses a conical beam (*α* > 10^−3^ rad) to produce diffraction disks, and the intensity distribution inside the disks allows unique determination of all the point groups and most space groups [4]. Because a selected area diffraction pattern can be recorded from almost every grain in a polycrystalline material, reciprocal lattices (≡crystal structures) and mutual crystal orientation relationships can be easily obtained. Therefore single crystal structural information can be obtained for many materials for which single crystals of the sizes suitable for x-ray or neutron diffraction are unavailable. Such materials include metastable or unstable phases, products of low temperature phase transitions, fine precipitates, nanosize particles etc.

**Fig. 2:**
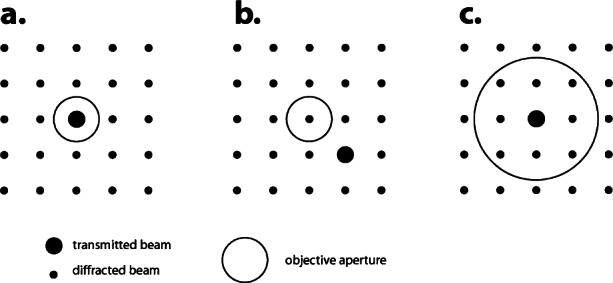
Three observation modes in electron microscope using an objective aperture. The center of the objective aperture is on the optical axis. (a) Bright-field method; (b) dark-field method; (c) high-resolution electron microscopy (axial illumination).

In order to investigate an electron microscope image, first the electron diffraction pattern is obtained. Then by passing the transmitted beam or one of the diffracted beams through a small objective aperture (positioned in the back focal plane) and changing lenses to the imaging mode, we can observe the image with enhanced contrast. When only the transmitted beam is used, the observation mode is called the bright-field method (accordingly a bright-field image), Fig. 2a. When one diffracted beam is selected (Fig. 2b), it is called the dark field method (and a dark field image). The contrast in these images is attributed to the change of the amplitude of either the transmitted beam or diffracted beam due to absorption and dynamic scattering in the specimens. Thus the image contrast is called the absorption-diffraction, or the amplitude contrast. Amplitude-contrast images are suitable to study mesoscopic microstructures, e.g., precipitates, lattice defects, interfaces, and domains. Both kinematic and dynamic scattering theories are developed to identify crystallographic details of these heterogeneities [2,3].

It is also possible to form electron microscope images by selecting more than two beams on the back focal plane using a large objective aperture, as shown in Fig. 2c. This observation mode is called high-resolution electron microscopy (HREM). The image results from the multiple beam interference (because of the differences of phase of the transmitted and diffracted beams) and is called the phase contrast image. For a very thin specimen and aberration-compensating condition of a microscope, the phase contrast corresponds closely to the projected potential of a structure. For a thicker specimen and less favorable conditions the phase contrast has to be compared with calculated images. Theory of dynamic scattering and phase contrast formation is now well developed for multislice and Bloch waves methods [5]. HREM can be used to determine an approximate structural model, with further refinement of the model using much higher resolution powder x-ray or neutron diffraction. However, the most powerful use of HREM is in determining disordered or defect structures. Many of the disordered structures are impossible either to detect or determine by other methods.

Other major advantages in using electron scattering for crystallographic studies is that the scattering cross section of matter for electrons is 10^3^ to 10^4^ larger than for x rays and neutrons, typical wavelengths (≈2 pm) are one hundredth of those for x rays and neutrons, and the electron beam can be focused to extremely fine probe sizes (≈1 nm) [2]. These characteristics mean that much smaller objects can be studied as single crystals with electrons than with other radiation sources. It also means a great sensitivity to small deviations from an average structure caused by ordering, structural distortions, short-range ordering, or presence of defects. Such changes often contribute either very weak superstructure reflections, or diffuse intensity, both of which are very difficult to detect by x-ray or neutron diffraction.

In addition, modern transmission electron microscopes provide a number of complementary capabilities known as analytical electron microscopy [6]. Different detectors analyze inelasticly scattered electrons (Electron Energy-Loss Spectroscopy, or EELS), excited electromagnetic waves (Energy Dispersion Spectroscopy, or EDS) and Z-contrast that provide information on chemical compositions and local atomic environments. Such information, when combined with elastic electron diffraction, is important in determining structural models, especially when a material consists of multiple phases.

In the following sections, various contributions of NBS/NIST researchers in the field of materials research with TEM as a central part of investigation are presented. The emphasis is on crystallographic aspects of the research. The presented contributions come mainly from the Materials Science and Engineering Laboratory.

## 2. Discovery of New Structures Using Selected Area and Convergent Beam Electron Diffraction

Starting in the early 1980s the Metallurgy Division of NBS was actively involved in studying the fundamentals of rapid solidification of a melt. In this process, materials (mostly metallic alloys) crystallize under very rapid cooling conditions (over 10^4^ °C/s). Such extreme conditions very often result in the formation of either new metastable or non-equilibrium crystalline or glassy structures. The rapid cooling also causes the formation of small-grain polycrystalline microstructures, the consequence of a high nucleation rate within the liquid. The combination of metastable (and therefore most probably unknown) structures with very small grain sizes makes such materials extremely difficult to study by x-ray diffraction, but very suitable for TEM.

A study of rapidly solidified Al-Mn alloys by Dan Shechtman resulted in one of the most important discoveries of modern crystallography—a quasiperiodic structure with icosahedral symmetry, thus including 5-fold, 3-fold, and 2-fold rotation axes of symmetry [7]. Such symmetry was inconsistent with the entire science of crystallography at that time. The icosahedral symmetry of the phase was demonstrated by carefully constructing a reciprocal lattice using a series of selected area electron diffraction. For the first time the existence of a well-ordered homogeneous (not twinned !) structure having symmetry elements incompatible with translational periodicity was shown. J. W. Cahn and D. Shechtman discuss the history of this remarkable discovery and its crystallographic aspects in a separate article in this issue.

**Fig. 3:**
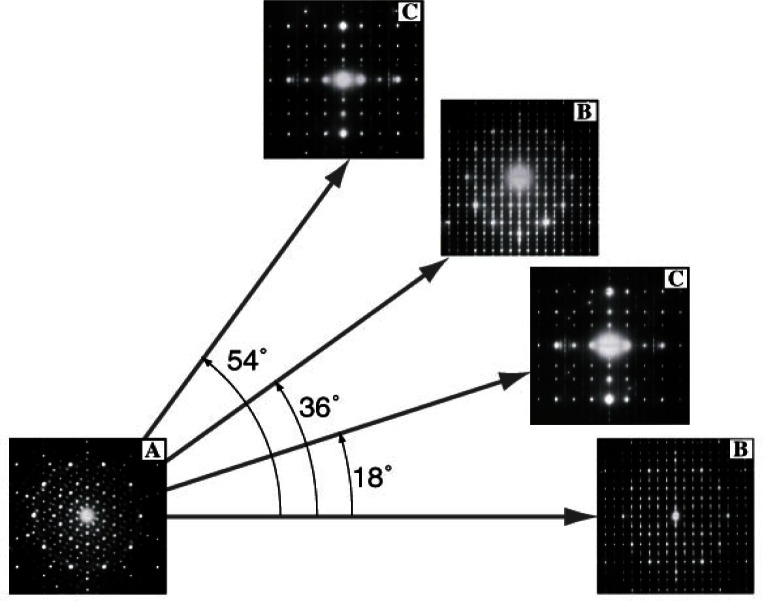
A series of SAD electron diffraction patterns obtained from the Al_78_Mn_22_ rapidly solidified alloy by tilting a single grain. Based on these patterns, a unique non-crystallographic 10-fold axis and a one-dimensional periodicity of the decagonal phase were established.

The discovery of the icosahedral phase triggered a period of very active research in the new field of quasicrystals. Many NIST researchers contributed actively in the early stages, and TEM played an important role in many aspects of this activity. Shortly after the Shechtman et al. publication, L. Bendersky discovered a different type of quasiperiodic structure—the decagonal phase with a 10-fold rotation axis, which has an apparent 10/mmm point group (Fig. 3) [8]. Electron diffraction analysis of this Al_80_Mn_20_ rapidly quenched alloy showed that the decagonal phase has a structure of two-dimensionally quasiperiodic layers, which are stacked periodically along the ten-fold axis, with a lattice parameter *c* = 1.24 nm. Shortly thereafter, a similar decagonal phase but with a different periodicity (*c* = 1.65 nm) was found in the Al-Pd system [8]. The importance of the discovery was not only discovery of a novel structure, but also demonstration of the general principles of quasiperiodicity. Since the discovery of the first quasiperiodic structures in Al-Mn alloys in 1984, enormous progress, both experimental and theoretical, has been made. Quasicrystalline phases have been found in more than hundred different metallic systems, and several quasicrystalline phases have been shown to be thermodynamically more stable than periodic crystals [9].

**Fig. 4:**
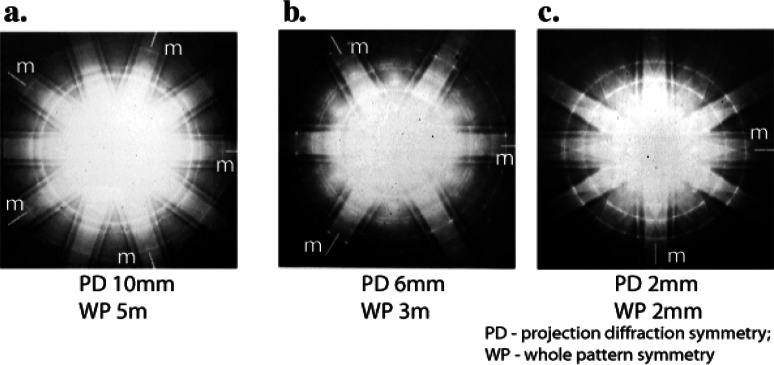
CBED patterns taken along (a) fivefold [1*τ*0], (b) threefold [111] and (c) twofold [001] orientations. The lines indicate the mirror planes (m).

**Fig. 5:**
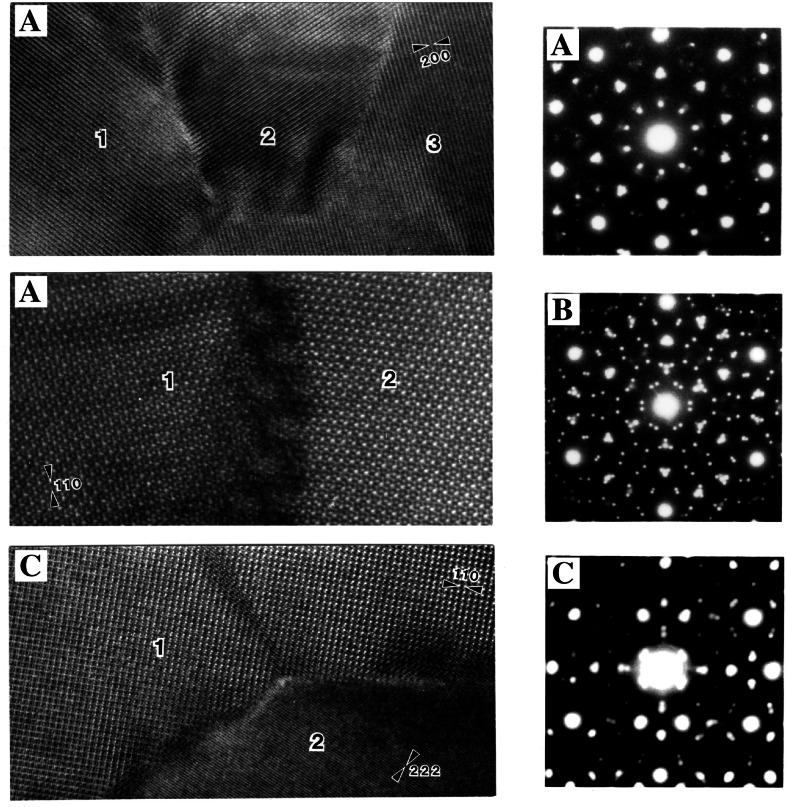
Polycrystalline aggregates of a cubic phase (*α*-Al_9_(Mn,Fe)_2_Si_2_) with their overall icosahedral symmetry were found in rapidly solidified Al_75_Mn_15−_*_x_*Fe*_x_* Si_10_. A, B, and C are high-resolution images of five orientational variants and corresponding SAD patterns in five-, three-, and two-fold orientation, respectively.

Among other significant discoveries at NIST associated with the new field of “quasi-crystallography” were:

- The first conclusive determination of the $m\bar{3}5$ point group for the icosahedral phase (for Al-38 %Mn-5 %Si (mass fraction) rapidly solidified alloy) [10]. Here the methods of convergent beam electron diffraction (CBED) were applied for the first time to a quasiperiodic structure. Fig. 4 shows an example of such CBED patterns from which the whole (3-dimensional) pattern symmetries of fivefold [1*τ*0], threefold [111] and twofold [001] orientations were derived (*τ*-irrational “golden mean” number).
- Polycrystalline aggregates of a cubic phase [*α*-Al_9_(Mn,Fe)_2_Si_2_] with an overall icosahedral symmetry were found in rapidly solidified Al_75_Mn_15−_*_x_*Fe*_x_*Si_10_ (*x* = 5 and 10) alloys [11]. Through a twinning operation, the cubic axes undergo five-fold rotation about irrational <1,*τ*,0> axes; however only five orientations occur among hundreds of crystals (Fig. 5). This is a special orientation relationship without any coincidence (or twin) lattice, and it is dictated by the non-crystallographic symmetry of a motif (in the case of the *α* phase—the 54-atom Mackay-icosahedron motif). The motifs are parallel throughout the entire polycrystalline aggregate, and the crystal axes change across grain boundaries. Based on this finding, the entire concept of twinning and special grain boundaries was re-examined. A new definition of special orientations, including hypertwins, based on reduction of the number of arithmetically independent lattice vectors was proposed. This new classification of special orientations within crystalline structures includes both old and new special orientations and can be easily interpreted in terms of quasilattices.
- The nucleation and growth properties of icosahedral and decagonal phases were examined. The study showed that dendritic Al-Mn icosahedral crystals grow along three-fold axes, and the decagonal phase nucleates epitaxially on the icosahedral phase, with coinciding five- and ten-fold axes [12]. F. Gayle gave beautiful images of the faceted icosahedral crystals in his study of an Al-Li-Cu alloy [13], which showed growth of icosahedral phase dendrites along 5-fold axes.
- TEM was used to study nucleation behavior of the icosahedral phase in submicron size droplets of Al-14 % Mn (atom fraction) produced by electrohydrodynamic atomization [14]. The icosahedral phase was found to nucleate very easily; this phenomenon was explained by the possible topological similarities of atomic packing in liquids and icosahedral quasicrystals. Observation of a metallic glass-like structure for the highest cooling rates was explained by the microquasicrystalline structure rather than by a conventional structure of frozen melt. Modeling of a x-ray diffraction spectra (obtained from a similar but vapor deposited structure of Al-Mn) [15] and analysis of heat evolution during DSC heating of as-deposited samples [16] proved the concept of microquasicrystalline state.

**Fig. 6:**
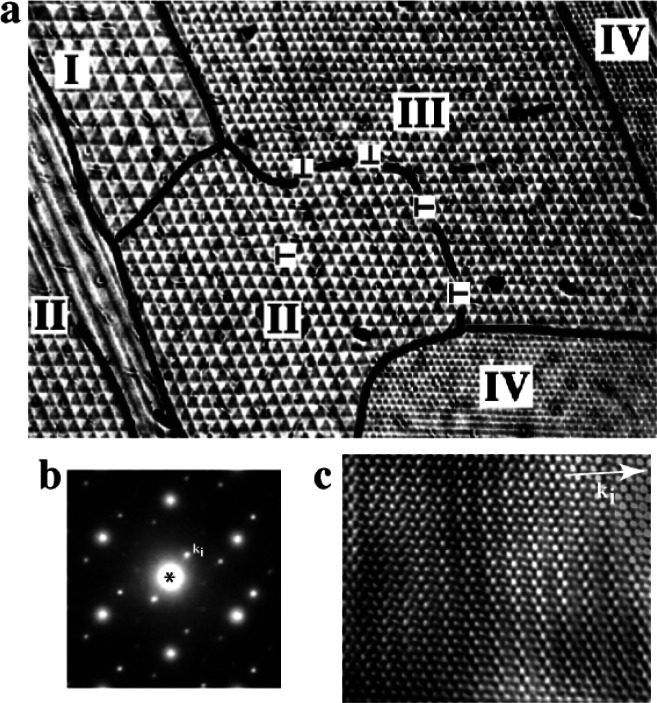
(a) Electron diffraction and (b) corresponding high-resolution image of the Zr_3_Rh_4_ compound having a basic rhombohedral structure and one-dimensional incommensurate modulation *k*_i_. (c) Optical image showing a regular array of triangular domains forming a macrolattice with defects. The domains are related to crystallographic variants (different directions of a *k*-vector) of the incommensurate phase.

Taking advantage of the strong scattering of electrons, the presence of incommensurate modulations has been established in several compounds. For example, in Zr_3_Rh_4_ (with a basic rhombohedral structure) one-dimensional modulations of atomic distortions was found. In this structure the modulation vector is normal to the *c*-axis and incommensurate both in its *k*-value and direction (Fig. 6) [17]. Further study of the Zr_3_(Rh_1−_*_x_*Pd*_x_*)_4_ alloys has revealed an intriguing correlation between the occurrence of the incommensurate modulation and magnetic and superconducting properties [18].

Incommensurate modulations were also found in a series of the layered Sr*_n_*(Nb,Ti)*_n_*O_3_*_n_*_+2_ compounds [19]. These structures are composed of pseudo-two-dimensional slabs of a distorted perovskite structure. The slabs are *n*-octahedra thick and extend parallel to the {110} perovskite plane. The compounds with *n* = 4, 5, 6, and 7 were observed to undergo a commensurate-incommensurate phase transition in the temperature range 150 °C to 250 °C. The wave-vector of the incommensurate modulation is parallel to [100] direction of the basic orthorhombic lattice. The incommensurate phase transition observed in several Sr*_n_*(Nb,Ti)*_n_*O_3_*_n_*_+2_ compounds were attributed to the structural distortion approximated by the alternating tilting of the (Ti,Nb)O_6_ octahedra, with amplitudes varying in the direction of the modulation.

Very different types of incommensurate modulation were recently found in Ruddlesden-Popper (*n* = 2) La_2−2_*_x_*Ca_1+2_*_x_*Mn_2_O_7_ compounds [20]. For the composition range 0.6 ≤ *x* ≤ 0.8, two sets of near-orthogonal incommensurate *k*-vectors normal to the tetragonal *c*-axis were identified by electron diffraction. High-resolution imaging confirmed the presence of the incommensurate two-dimensional lattice. The lattice can be approximated with *a*_2D_ = 5*a*_p_ (*a*_p_—perovskite unit cell). A model based on the previously unobserved type of 4:1 charge/orbital ordering between the Mn^4+^ and Mn^3+^ cations was suggested. Investigation of the magnetic and electric properties of the compounds also indicates the presence of the charge ordering.

## 3. Structural High-Resolution Microscopy

High-resolution electron microscopy techniques were developed in the early 1970s for imaging structures of inorganic crystals at the unit cell level and their extended defects. In these early works the researchers used electron microscopes with rather limited resolution, and the approach was mainly to identify one-dimensional structural sequences. Robert S. Roth was the first at NBS to realize the opportunities of using TEM structural imaging to study structures of complex oxides, and in particular “solid solutions,” which in fact occurred by intergrowth of layers of closely related structures/phases. In the 70s he actively collaborated with the pioneers in multi-beam structural imaging, J. Allpress from Australia and S. Iijima from Japan (at that time with Arizona State University). In the 5th Materials Research Symposium sponsored by NBS and organized by R. S. Roth and S. J. Schneider, Jr., many of the publications were dedicated to resolving structural issues with the help of novel multi-beam imaging.

**Fig. 7:**
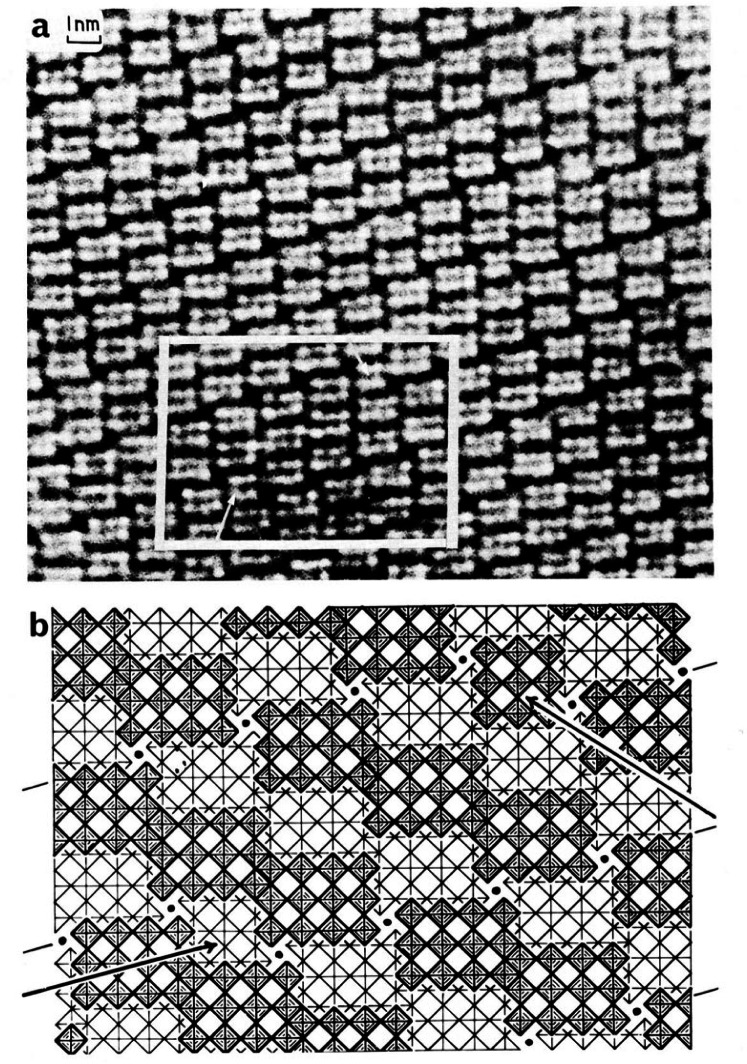
(a) High-resolution two-dimensional lattice image from a fragment of TiO_2_:7Nb_2_O_5_, showing several displacements associated with the presence of isolated 3 × 3 blocks (arrowed) in the matrix of 4 × 3 blocks. (b) Idealized model of the area outlined in (a). The crystallographic shear (CS) planes (containing tetrahedral metal atoms) are marked. They suffer a displacement at the points where 3 × 3 blocks (arrowed) occur.

Collaboration between S. Iijima and R. S. Roth resulted in the publication of one of the first high-resolution two-dimensional images where the intergrowth of 4 × 4, 4 × 3, and 3 × 3 blocks in TiO_2_·7Nb_2_O_5_ was imaged, thus demonstrating the possibility of solving complex structures directly (Fig. 7, adapted from [21]). This and similar efforts were essential in establishing the important structural principles of crystallographic shear and Wadsley defects (coherent intergrowth of one member of the structural family with another). Within the systems studied by Roth were polymorphs of ZrO_2_:12Nb_2_O_5_ [22], off-stoichiometric *x*Nb_2_O_5_·*y*WO_3_ compounds [23] and mixed-block structures of Rb_2_O-Ta_2_O_5_, Rb_2_O-Nb_2_O_5_, and K_2_O-Ta_2_O_5_ [24].

In the 1980s there was an increased interest in high-quality microwave dielectrics with a high dielectric constant (>30), low losses and near-zero temperature coefficient of the dielectric constant. At that time, the most acceptable materials were barium titanates. R. S. Roth and his post-doc P. K. Davies studied structural variations of these compounds by using the newly installed at NIST Philips 430 microscope^1^ with 0.23 nm point-to-point resolution [25]. Using structural imaging, they ascertained the mechanisms of intergrowth defect formation in two important compounds, Ba_2_Ti_9_O_20_ and BaTi_5_O_11_. They found that the most prevalent defect was formation of a new triclinic polytype, with an ionic arrangement closely related to that in the accepted structure. Stoichiometric defects with considerable microtwinning and formation of face-sharing octahedra were also observed, while nonstoichiometric defect formation was minimal. TEM structural investigations of microwave dielectrics continued in 1990s, in conjunction with the phase diagram studies of these materials in the Ceramics Division of NIST by T. Vanderah and R. Roth.

With the acquisition of the ultra-high resolution JEOL3010 microscope by the Materials Science and Engineering Laboratory (MSEL) of NIST in the 1990s, investigation of structures and defects by high-resolution imaging became routine. In the study of newly discovered structures, often the approach was to develop an approximate model based on experimental high-resolution images and electron diffraction, and then to work out image simulations compatible with the experimental images, and finally to refine the model using higher resolution x-ray or neutron powder diffraction.

**Fig. 8:**
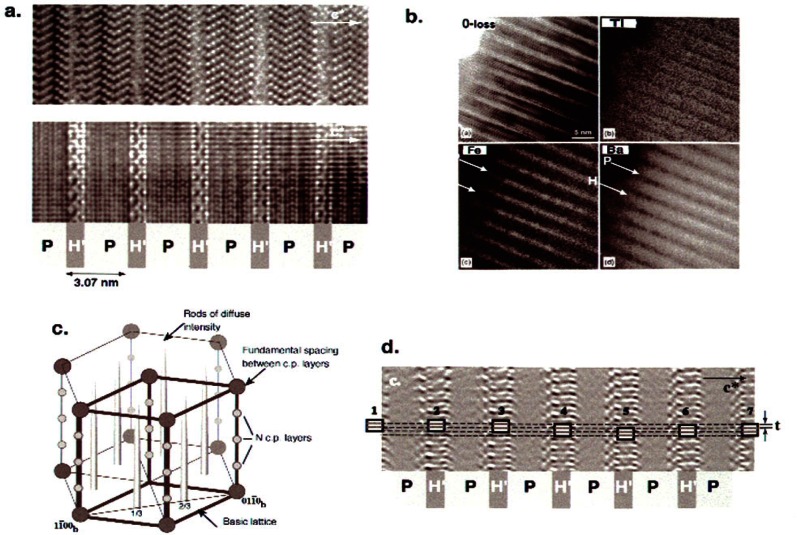
(a) HRTEM images of the M phase taken in orientations corresponding to $\left[11\bar{2}0\right]$ (upper micrograph) and $\left[1\bar{1}00\right]$ (lower micrograph) zone axes of the hexagonal Ba(Ti,Fe)O_3_ layer. On the background the yellow and pink stripes correspond to P (perovskite) and H-type (magnetite) slabs, respectively. (b) Composional maps of the M phase (in $\left[11\bar{2}0\right]$ zone axis orientaion) obtained with Ti L_2,3_, Ba M_4,5_, and Fe L_2,3_ edges. The maps show the Ti composition is relatively uniform throughout the crystals whereas the Fe elemental distribution reveals an enhancement corresponding to the H slabs. Similarly, the Ba distribution is decreased in the H slabs relative to the P slabs. (c) A schematic drawing of a reciprocal lattice of the compounds showing the typical rods of continuous intensity in the *c*-direction. (d) The image obtained by inverse FFT shows only the H slabs. Rectangles correspond to a characteristic structural element of the H slab and emphasize random shift of the H slabs.

A very intriguing and new structural architecture has been recently discovered with the help of high-resolution imaging for a group of six new ferrimagnetic phases in the BaO:TiO_2_:Fe_2_O_3_ system [26]. The compounds were proven to belong to a class of well-ordered intergrowth structures. While in general the structures exhibit a close-packed framework of [O + (Ba/O)] layers (with stacking sequences different for different phases), two types of alternating structural two-dimensional slabs were identified. The first has a structure of the hexagonal *h*-BaTiO_3_ and the second—an Fe-rich, spinel-like structure. Fig. 8a gives an example of high-resolution imaging of such structure (observed for the M phase). Energy-filtered mapping using EELS was used to establish the compositional distribution of Fe, Ti, and Ba (Fig. 8b) [27]. The strongly heterogeneous distribution of Fe suggests that all six phases can be considered as self-assembled magnetic multilayer structures, with potentially intriguing physical properties. One-dimensional structural disorder was observed typically for the phases (Fig. 8c) and identified as resulting from a poor spatial correlation (translation within a closed-packed plane) between the h-BaTiO_3_ blocks (Fig. 8d). The disorder was responsible for the difficulty of determining the structures using x-ray diffraction, even with the use of a single crystal.

**Fig. 9:**
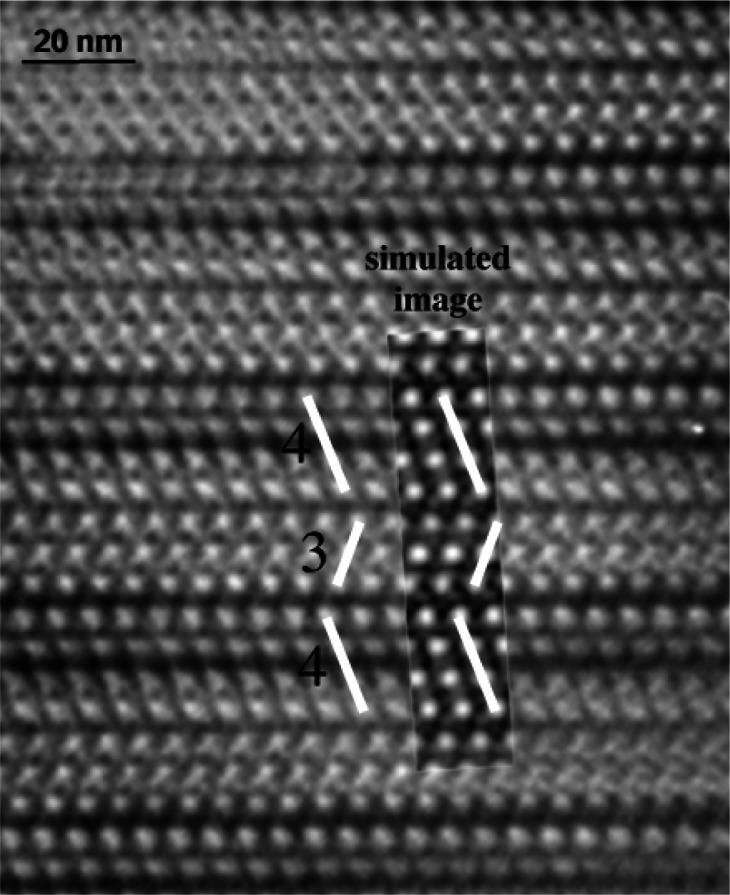
High-resolution TEM image for 7M-Ca_2_Ta_2_O_7_ viewed along [110] and the corresponding simulated phase-contrast image (inset). The simulated image was obtained for 35 nm thickness, −50 nm defocus value and the 10 nm^−1^ aperture. The 4- and 3-layers structural blocks are emphasized.

A structural study of the microwave dielectric calcium tantalate, Ca_2_Ta_2_O_7_, showed diffraction patterns displaying strong subcell characteristics of the fluorite structure. The complex distributions of superlattice reflections depend on the nature and concentration of the dopant and on the reaction temperature. Polytypes with *n* = 3, 4, 5, 6, and 7 were prepared as single crystals by the flux method [28]. High-resolution electron microscope images were used to determine a characteristic structural feature of the polytypes (Fig. 9). The major feature was a fluorite-type cation array, which is periodically twinned on (111)_fluorite_ to give mixed cubic-hexagonal stacking of the cation layers. The basic repeating units are slabs of different thicknesses comprising a Ca_3_Ta layer and a Ta_3_Ca layer. Single crystal x-ray and powder x-ray and neutron data were used to determine an exact structure of the polytypes. For *n* = 3, the structure is the 3-block trigonal weberite P3_1_21 (3T) [28]. In the 7M polytype the *h*-stacked Ta_3_Ca layers divide the structure into two blocks of *c*-stacked layers which contain 3 and 4 repeating units and can be thus designated as 3M and 4M blocks. The stacking sequence of metal atom layers in the 7M polytype is *hccccchccccccch*… For the 5M polytype, the *h*-stacked layers divide the structure into two 2M and 3M blocks. The metal atom layer stacking sequence is then *hccchccccch*… [29].

## 4. Study of Phase Transitions

The essential feature of a structural phase transition is the change of symmetry. Most often it is a low temperature (room temperature) phase that is studied, which has a lower symmetry than the high temperature phase(s). Its space group often is a subgroup of that of the high temperature phase. Therefore, a single crystal (grain) of the high temperature phase at low temperature is subdivided into a number of symmetry-related domains, or structural variants. The variants are either rotational (twins) or translational (anti-phase domains, or APDs), and separated from each other by specific interfaces. Knowledge of the crystallographic nature of the interfaces and the structure of the low temperature phase will often allow prediction of the structure of high temperature phases and possible intermediate phases [30,31]. TEM direct-space imaging (amplitude contrast), in combination with SAD and CB electron diffraction, is the most suitable technique for this approach. For very small domain structures, high-resolution imaging and Fast-Fourier Transform are most appropriate.

**Fig. 10:**
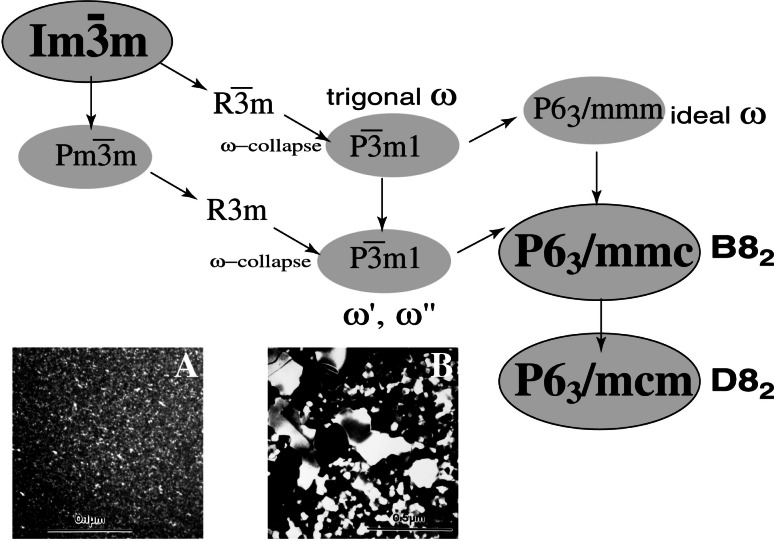
A sequence of *ω*-related phases formed from the high temperature B2 (ordered BCC) phase in the Ti_4_AI_3_Nb alloy is summarized in the form of maximal group-subgroup relationship. Dark field images A and B show microstructures of (A) metastable *ω*′ phase and (B) a mixture of *ω*″ and B8_2_ phases.

In the early 1990s the interest in a new generation of high-temperature materials for aerospace applications led scientists from the Metallurgy Division of NIST to study intermetallic compounds, in particular the Ti-Al-Nb alloys. The use of TEM was extremely helpful in understanding the complexity of phase transformations occurring in these materials. The transformations in the Ti-Al-Nb system include (1) a martensitic-like transition from the high-temperature BCC phase, with formation of a Widmanstatten-type lamellar structure (an HCP-to-FCC transition plus ordering) and omega-type displacements [32]. Study of the Ti_2_AlNb and Ti_4_AlNb_3_ alloys showed the presence of two phases: the Ti/Nb-rich b.c.c. phase and the ternary orthorhombic Ti_2_AlNb phase [33]. Depending on the alloy composition, one of the phases was observed as a precipitate with plate morphology in the matrix of the other phase. For the Ti_4_Al_3_Nb alloy, formation of a sequence of metastable *ω*-related phases from the B2 (ordered BCC) phase was established and summarized in the form of maximal group-subgroup relationship (Fig. 10) [34]. An equilibrium low temperature phase has the B8_2_ structure (and triple-cell D8_2_ structure for the more Nb-rich compositions). The metastable phase and both B8_2_ and D8_2_ are structurally related to the B2 phase by displacement (collapse) of every two (out of three) (111) planes. The *ω*-type structures were verified by means of transmission electron microscopy and by single crystal x-ray diffraction.

**Fig. 11:**
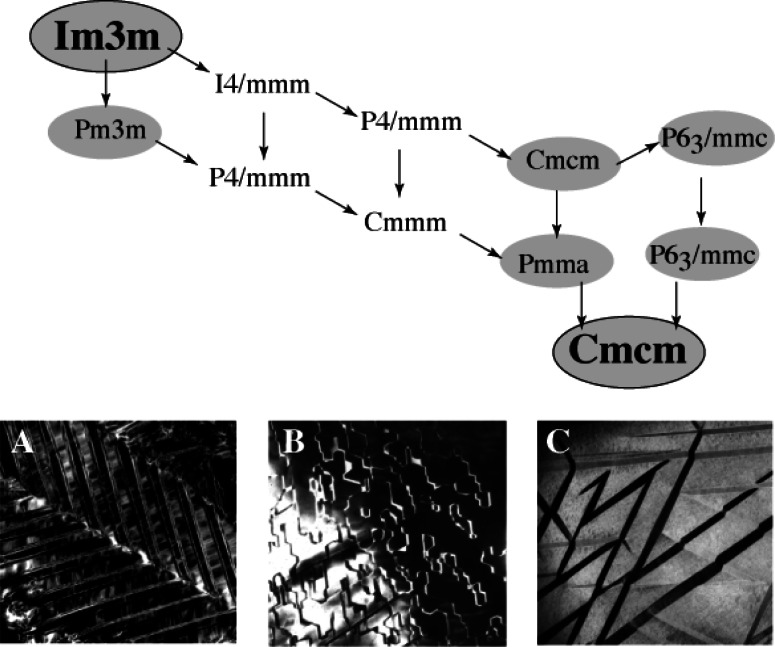
A sequence of phases formed from the high temperature B2 (ordered BCC) Ti-Al-Nb phase is summarized in the form of maximal group-subgroup relationship. (A) Ti_2_AINb alloy, rotational domains of the O phase accommodated in the form of hierarchical twin structure; (B) Ti_2_AINb alloy, interfaces between translational domain formed in the *Pm*3*m*(B2)-to-*Pmma*(B19) transition; (C) two-phase mixture of DO_19_ (matrix) and O-phase (precipitates).

For Ti-25 %Al-12.5 %Nb (atom fraction), the transformation path from high temperatures includes the formation of the intermediate hexagonal DO_19_ phase, and subsequent formation of the low-temperature orthorhombic O (Ti_2_AlNb) phase. For alloys close to Ti-25 %Al-25 %Nb (atom fraction), the path involves an intermediate Bl9 structure and subsequent formation of a translational domain structure of the O phase [35]. The experimental results were summarized as a sequence of crystallographic structural relationships developed from subgroup symmetry relations [36] (Fig. 11). Symmetry elements lost in each step of the sequence determine the possibilities for variants of the low symmetry phase and domains with characteristic interfaces that can be present in the microstructure. Fig. 11 gives three examples of such domain: (A) Ti_2_AlNb alloy, rotational domains of the O phase accommodated in the form of a hierarchical twin structure; (B) Ti_2_AlNb alloy, interfaces between translational domains formed in the course of the $Pm\bar{3}m(B2)$-to-*Pmm*a(B19) transition; (C) a two-phase mixture of DO_19_ (a matrix) and three-variants of O-phase (precipitates). The orientation of inter-domain interfaces is determined by the requirement for a strain-free interface (as in Fig. 11A and C). A structure of the O-phase (an important ternary phase, currently a base for new high-temperature composites) was refined using the preliminary model derived from the TEM studies and powder neutron diffraction data [37].

**Fig. 12:**
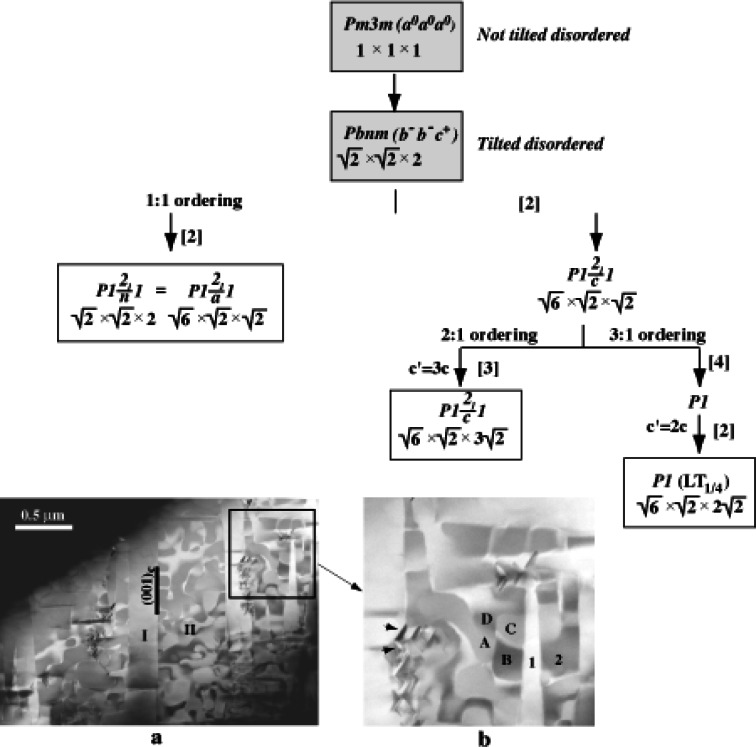
Top. Symmetry tree describing group/subgroup relations between the three Ca_4_Nb_2_O_9_ polymorphs with distinct (1:1, 1:2, and 1:3) arrangements of the B-cations; the numbers in brackets indicate the number of crystallographic variants generated by the corresponding minimal symmetry reductions. Since all three phases feature similar *b*^−^*b*^−^*c*^+^ octahedral tilting, their space groups are subgroups of *Pbnm*, which describes a disordered perovskite structure with the same tilting. Bottom: a-Dark-field image of the single grain containing the metastable 1:3 ordered Ca_4_Nb_2_O_9_ polymorph. b-Magnified view of the area outlined by rectangular in (a). Twin-type domains **I** and **II** in the area labelled a are related to the *Pm*3*m*→*Pbnm* octahedral tilting transition. The domains exhibit a substructure consisting of both rotational (1 and 2 in the area labelled b) and translational (A, B, C, and D) domains. The domains 1 and 2 can be described by the *Pbnm*→*P*1 symmetry reduction, while the domains A, B, C, and D can be accounted for by the lost of the *c*-glide plane (*P*2_1_/*c*→*P*1) and doubling of the *c* lattice parameter. Additionally precipitates of the 1:2 ordered polymorph nucleated in the 1:3 ordered matrix are seen in b (indicated by arrows).

The approach of the maximal group-subgroup relationship was also applied to phase transformations in other systems, and in particular to transformations between ordered polymorphs of the microwave dielectric Ca(Ca_1/3_Nb_2/3_)O_3_ [38] and Ca(Ca_1/3_Nb_2/3_)O_3_-CaTiO_3_ [39]. Four Ca_4_Nb_2_O_9_ polymorphs with perovskite-related structures and different arrangements of B-site cations were identified. Ordering of the B-cations (Ca^2+^ and Nb^5+^) in this system is combined with displacement of oxygen ions by octahedral tilting. Three of the polymorphs have 1:1 (monoclinic *P*2_1_/*c*), 2:1 (monoclinic *P*2_1_/*c*) and 3:1 (triclinic *P*1) ordering. The 1:1 ordered structure features alternating {111}_c_ planes occupied exclusively by Nb, and the occupied by a disordered mixture of Ca and the remaining Nb cations, while the 1:2 ordered polymorph exhibits {CaNbNb…} sequence of the {111}_c_ B-cation planes. The metastable 3:1 structure is derived from the 1:1 ordered structure by a partial ordering of Ca and Nb cations on those B-sites which were occupied by a random Ca/Nb mixture in the 1:1 ordered array; the resulting ordered arrangement can be viewed as 1:3 type. The models were subsequently validated by Rietveld refinements using combined x-ray and neutron powder diffraction data [40]. The relationship between the polymorphs in the form of maximal subgroup relationship was derived from the complex hierarchy of interfaces observed by TEM (Fig. 12). The space groups of the ordered Ca_4_Nb_2_O_9_ polymorphs (deduced from electron diffraction data) do not exhibit group/subgroup relations, and, therefore, phase transitions between them must be first-order transitions.

Different problems related to the microstructural formation were solved using conventional TEM. For example, in the study of Diffusion-Induced Grain Boundary Migration (DIGM) phenomena, the effect of diffusion of Cr_2_O_3_ into polycrystalline Al_2_O_3_ down grain boundaries and concomitant generation of local stresses due to the change of lattice parameter were studied [41]. Relief of these stresses occurred by grain boundary migration and the generation of misfit dislocations. When these dislocations were observed using bright and dark field transmission electron microscopy, a good correlation was found between the dislocation spacing and the chromium concentration in the alloyed regions. Another problem related to the plastic deformation of A1_2_O_3_ by slip and twinning was investigated by examining the regions surrounding a microhardness indentation using TEM [42]. The results establish: (1) the occurrence of pyramidal slip on $\{11\bar{2}3\}\langle 1\bar{1}00\rangle$, and (2) the nature of basal twins in this material. The observations on basal twins, in particular, have led to a completely different description for the twinning process. In the study of important Ti-Al aerospace alloys, the morphology of discontinuous coarsening in the Ti_3_Al (*α*_2_) / TiAl (*γ*) fully lamellar structure was examined [43]. Three morphologies were observed in discontinuously coarsened lamellar structures (secondary lamellae). Type-(I) lamellae have the low energy habit plane as their lamellar interfaces, and have the same lamellar direction as the original primary lamellae. Type-(II) lamellae have the same crystallographic orientation of *α*_2_ plates as that in the original primary lamellae, but have a different lamellar direction from the original primary lamellae, and have irregular faceted lamellar interfaces. Type-(III) lamellae have a different lamellar direction and a different crystallographic orientation of the *α*_2_ plates from that in the original primary lamellae, but have the low energy habit plane as their lamellar interfaces. The growth kinetics of these three types of lamellae were analyzed by modifying the Livingston and Cahn treatment in order to obtain the dependence of the secondary lamellar morphologies on misorientation between the penetrated primary lamellae, the advancing secondary lamellae and the lamellar colony boundaries.

**Fig. 13:**
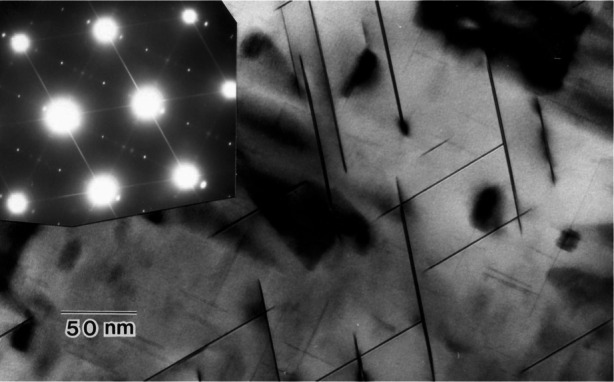
This ultra high strength aluminum alloy is strengthened by a mix of precipitates, including the prominent T1-Al_2_CuLi platelets lying on matrix {111} planes, and platelike *θ*′ (on {100} planes) and lath S′ precipitates (on {210} planes), identified by the diffraction pattern.

Precipitation of second phases in aluminum alloys presents another challenging application for electron diffraction due to the small dimensions and multiple variants of the precipitates, which are most often metastable phases in commercial alloys and tempers. In the late 1980s the Metallurgy Division had a collaborative effort with Martin Marietta Laboratory to determine the origins of the ultra-high strength in Weldalite aluminum alloys, later used to build the Space Shuttle External Tank to reduce launch weight. This novel aluminum alloy was primarily strengthened with 1.2 % Li and 4.0 % Cu (mass fraction). Silver and magnesium were added, 0.4 % each, to aid precipitation of second phases, and 0.1 % Zr was added to inhibit recrystallization. TEM imaging and diffraction was required to sort out the complex microstructure responsible for the high strength of the alloy. Our microstructural studies established that the Weldalite alloys are strengthened primarily by the equilibrium T_1_-Al_2_CuLi phase, in contrast to other commercial aluminum alloys, which are strengthened by metastable precipitate phases. In addition to T_1_, additional strengthening arises from 3 other lath or plate-like precipitates (S′-Al_2_CuMg, *η*′-Al_2_Cu and an unknown phase), all lying on distinct habit planes in the matrix (Fig. 13) [44]. When *α*′, or Al_3_(Zr,X), which inhibits grain boundary motion, is included, it appears that this alloy in the highest strength condition may represent a metastable equilibrium between six phases.

## 5. Conclusions

The NBS/NIST Materials Science and Engineering Laboratory has a history of discovery of new phases and classes of crystallographic structures through transmission electron microscopy and electron diffraction. The power of electron diffraction has allowed the determination of structural details, both in perfect crystals and in defect structures, which are too subtle for x-ray or neutron diffraction techniques. Research within MSEL has lead to discoveries ranging from entirely new classes of crystallography and crystal defects to advancement of the understanding of commercial aerospace alloys.

## Footnotes

1 Certain commercial equipment, instruments, or materials are identified in this paper to foster understanding. Such identification does not imply recommendation or endorsement by the National Institute of Standards and Technology, nor does it imply that the materials or equipment identified are necessarily the best available for the purpose.
